# Surfactant concentration modulates the motion and placement of microparticles in an inhomogeneous electric field†

**DOI:** 10.1039/d0ra00703j

**Published:** 2020-03-02

**Authors:** Marcos K. Masukawa, Masayuki Hayakawa, Masahiro Takinoue

**Affiliations:** Department of Computer Science, Tokyo Institute of Technology 4259 Nagatsuta-cho, Midori-ku Yokohama Kanagawa 226-8502 Japan; Department of Computational Intelligence and Systems Science, School of Computing, Tokyo Institute of Technology 4259 Nagatsuta-cho, Midori-ku Yokohama Kanagawa 226-8502 Japan takinoue@c.titech.ac.jp; RIKEN Center for Biosystems Dynamics Research Kobe Hyogo 650-0047 Japan

## Abstract

This study examined the effects of surfactants on the motion and positioning of microparticles in an inhomogeneous electric field. The microparticles were suspended in oil with a surfactant and the electric field was generated using sawtooth-patterned electrodes. The microparticles were trapped, oscillating, or attached to the electrodes. The proportion of microparticles in each state was defined by the concentration of surfactant and the voltage applied to the electrodes. Based on the trajectory of the microparticles in the electric field, we developed a new physical model in which the surfactant adsorbed on the microparticles allowed the microparticles to be charged by contact with the electrodes, with either positive or negative charges, while the non-adsorbed surfactant micellizing in the oil contributed to charge relaxation. A simulation based on this model showed that the charging and charge relaxation, as modulated by the surfactant concentration, can explain the trajectories and proportion of the trapped, oscillating, and attached microparticles. These results will be useful for the development of novel self-assembly and transport technologies and colloids sensitive to electricity.

## Abstract 2

This study revealed that surfactant micelles played two roles for microparticle motions in an inhomogeneous electric field (trapping, oscillating, or attachment to electrodes): particle charging and charge relaxation, depending on its concentration.

## Graphical abstract

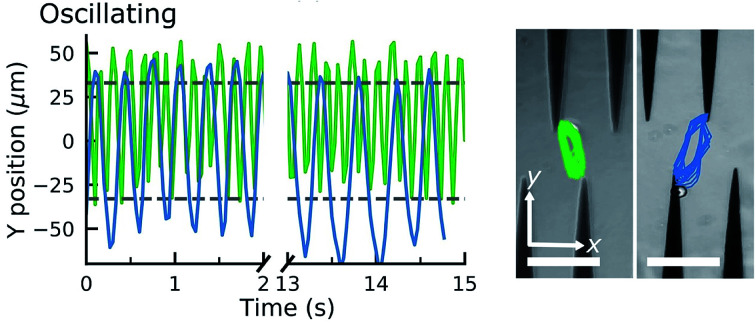

## Introduction

Techniques for manipulating microparticles are important in physical, chemical, and biological research.^1^ Fundamentally, the ability to control small particles in small volumes can help elucidate the mechanisms that operate at the μm scale. From a practical point of view, these mechanisms can be explored to build sensors and actuators—thereby extending the capabilities of microfluidic devices and display technologies and bridging the macro- and nanoscale.^2^

Researchers have used optical tweezers,^3^ surface acoustic waves,^4^ chemical gradients,^5^ and magnetic^6^ and electric fields,^7–14^ among other non-contact methods, to manipulate microparticles. Electric fields represent an attractive method for controlling microparticles in particular. For instance, electrodes can be designed to produce specific electric fields that can be quickly modulated *via* changes in the frequency and amplitude of the applied voltage.^15^ Microparticle movement in an electric field is often referred to as electrokinetics, and distinct mechanisms govern the interactions of microparticles with electric fields for displacement in a controlled manner.^16,17^ Examples include dielectrophoresis *via* the application of an inhomogeneous electric field^11,15,18,19^ and contact charge electrophoresis, when the particle charge is modified by contact with a charged object.^9,10,20^

To control a particle using an electric field, easily modified electric and dielectric properties of the particle and surrounding medium are desirable. Surfactants can modify the medium conductivity^21–24^ and charge microparticles suspended in apolar liquids.^24–32^ Although not fully understood, the modification of electrical properties of apolar colloids by surfactant addition is of great importance in industry as it is used to control the electrical properties of ink in printing processes.^21^ This is also used to prepare electrophoretic ink displays to control the position of the pigment microparticles^8^ and to set their position in an organized manner to form colloidal crystals,^33^ which can be used as photonic materials. Therefore, significant interest has been generated in the chemical synthesis of novel surfactants suitable for particle control,^27^ to clarify the role of surfactants in electrokinetics and extend its applications. However, the effect of surfactant concentration on the electrokinetics of microparticles remains unclear due to the challenges of producing a model that considers the surfactant concentration effect on both contact charge electrophoresis and charge relaxation. Such a model would be useful to predict the ideal surfactant concentration for the manipulation of microparticles by electric fields.

Herein, the effect of concentration of a neutral surfactant on microparticles suspended in an apolar liquid subject to an inhomogeneous electric field was studied. Based on the experimental observations, a model of particle charging and charge relaxation modulated by the surfactant for definition of displacement and position of the microparticles was developed and investigated.

## Materials and methods

### Sample preparation of microparticle suspension in oil with a surfactant

Liquid paraffin (Wako, 128-04375) was used as an oil phase, Sorbitan Monooleate (Span 80) (TCI chemicals, S0060) as a neutral surfactant, and polystyrene microbeads 20 μm in diameter (Micromod, 01-00-204) as microparticles. A stock solution was prepared by vortexing liquid paraffin and 0.0001% (w/w) Span 80, sonicating the mixture at 45 °C for 1 h and adding microparticles. A low concentration of microparticles, 0.01% (w/w), was used to minimize the interaction between microparticles. The stock was vortexed and sonicated again at 45 °C for 1 h. Afterwards, microparticle suspensions with different concentrations of surfactant were prepared by adding Span 80 to the stock solution, vortexing, sonicating at 45 °C for 1 h, and leaving the mixture to equilibrate for 1 h. Suspensions of microparticles were prepared in liquid paraffin with Span 80 concentrations ranging from 0.0001 to 5% (w/w).

### Sample preparation of the fluorescent reverse micelles

Reverse micelles of Span 80 aggregates containing fluorescein were prepared following the protocol described by Anton *et al.* (2011).^34^ First, a saturating amount of fluorescein (Sigma-Aldrich, F6377-100G) was added to liquid paraffin containing 0.1% (w/w) Span 80. The dispersion was then vortexed and sonicated at 45 °C. To remove fluorescein crystals, the dispersion was centrifuged for 10 min at 2000 × *g* and the supernatant was collected, with the process being repeated three times. Afterwards, the fluorescent dispersion of Span 80 was diluted to 0.005% (w/w) and the microparticles were added. The samples were subsequently vortexed and sonicated at 45 °C for 1 h. To prepare the fluorescent samples with different surfactant concentrations, Span 80 was added without fluorescein to the desired concentration. Fluorescent samples in concentrations of 0.005% (w/w) (no additional surfactant), 0.05% (w/w), 0.5% (w/w), and 5% (w/w) were prepared.

### Microelectrode fabrication

**Fig. 1:**
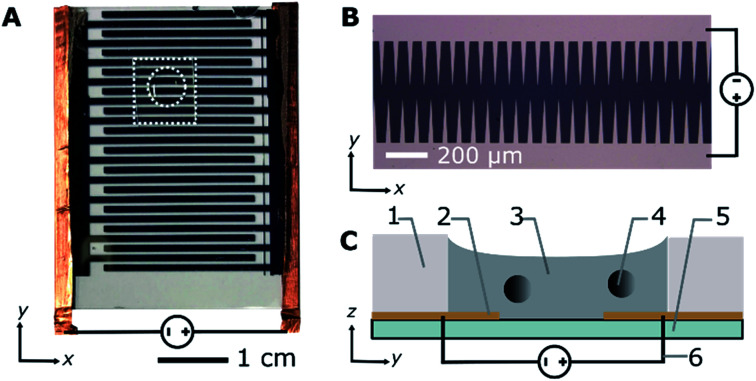
Experimental set-up. (A) Photograph of the device from the top, dashed lines indicate the gasket used to contain the sample. (B) Photograph of electrodes from the top against a dark background. (C) Illustration of side view. (1) PDMS gasket; (2) electrode with sawtooth edge; (3) liquid paraffin with Span 80; (4) polystyrene microparticle; (5) glass slide; (6) direct current voltage.

Interdigitated microelectrodes with sawtooth edges and a 70 μm gap between the teeth (Fig. 1A and B) were prepared using the lift-off method.^35^ Briefly, a S1818G photoresist (Microchem) was spin coated onto a micro cover glass No. 5 (Matsunami) treated with plasma oxidation (90 s on an ion bombarder, Vacuum Device Co., Ltd) and silylated with hexamethyldisilazane (HMDS, Wako AWK3814) *via* vapour phase deposition.^36^ The photoresist was spin coated at a maximum spin frequency of 3000 rpm for 30 s (Opticoat SpinCoater, Mikasa). Subsequently, the slide was pre-baked for 1 min at 115 °C, cooled to room temperature, and exposed using a maskless pattern generator with resolution of 3 μm (μPG 101, Heidelberg Instruments; laser wavelength, 375 nm). Afterwards, the photoresist was developed with tetramethylammonium hydroxide solution 2.38% (OFPR-NMD-3, Tokyo Ohka Kogyo Co., Ltd.) and cleaned with isopropyl alcohol (IPA, Kanto Chemical Co. Inc. JIS K8839). The developed slide was then coated sequentially with chromium and gold using a metal evaporator (VE2012 TMP vacuum evaporator, Vacuum Device Co., Ltd). Finally, the undeveloped photoresist was removed with acetone (Wako, DSG4138), revealing a sawtooth pattern. ESI Method 1† contains additional details regarding the sample preparation.

### Microscopic measurements

To track the microparticle position, 12 μL samples were placed directly on top of the microelectrodes and placed in a polydimethylsiloxane (PDMS) gasket with a 5 mm diameter (Fig. 1A and C). Direct current (DC) voltages of 100, 200, and 300 V were sequentially applied to the electrodes using a DC power supply (GWInstek, GPR30H10D). The measurements were performed in triplicate for all voltages and surfactant concentrations and, on average, the trajectory of 51 ± 21 microparticles per surfactant concentration per voltage were analysed for each experiment. The position of the microparticles was recorded using a phase contrast microscope (Olympus, CKX41) and digital camera (Canon, EOS60D). From the recorded videos, the positions of the individual microparticles were tracked using custom image analysis software and the Python package Trackpy^37^ and the motion patterns of the beads were classified (ESI Method 2†). Fluorescent samples were used to observe the location of the Span 80 aggregates and measure the ratio of the dispersed to adsorbed surfactant on the microparticles (ESI Method 3†). A fluorescence microscope (Olympus, IX71) equipped with a sCMOS camera (Andor, Zyla) was used to observe the fluorescent samples. The microscope was equipped with a mercury lamp source, mirror unit with 470–490 nm band-pass excitation filter, 505 nm dichroic mirror, and 510–550 nm band-pass emission filter (Olympus, NIBA).

### Numerical simulations

The finite element software COMSOL Multiphysics (COMSOL Inc., v4.3), was used to calculate the electric and dielectric fields generated by the saw tooth electrodes at 200 V. Using the fields, the trajectory of 500 microparticles with random initial conditions under 12 different surfactant concentrations were monitored, totalling 6000 simulated trajectories. For the simulation, a custom Python script with the Runge–Kutta 4^th^ order was used for numerical integration. After simulation, the robustness of the obtained results was determined by comparing 10 random subsets of trajectories. The theory for the simulation is discussed in the Results and ESI Discussions 1 and 2,† while the choice of parameters is discussed in the ESI Note 1.†

## Results and discussion

**Fig. 2:**
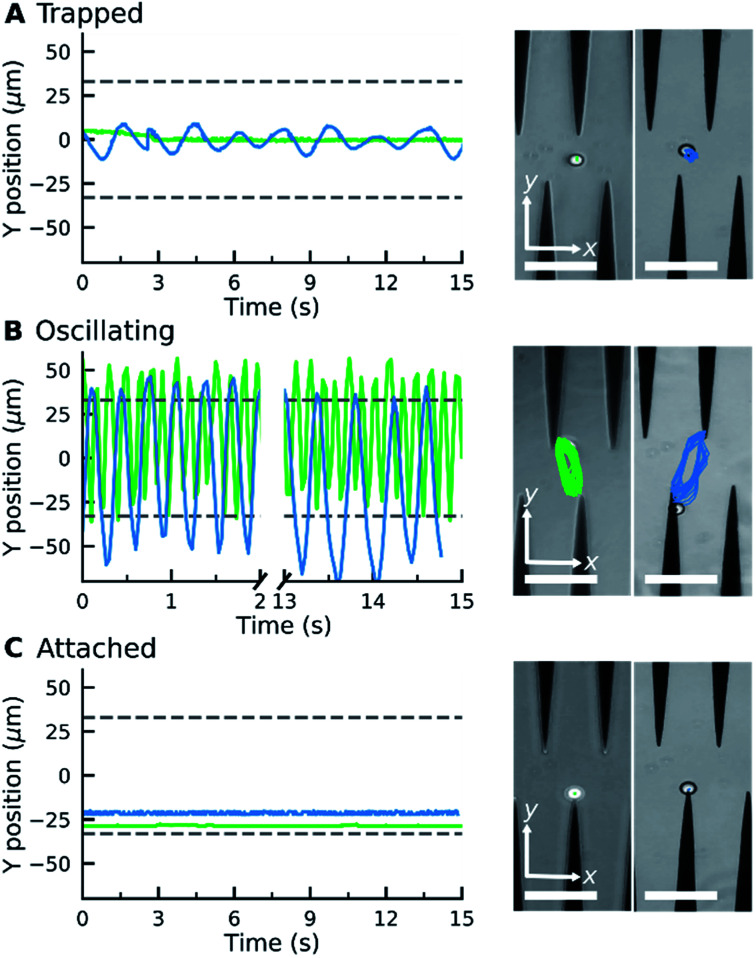
Microparticle classification into three patterns of motion based on microparticle trajectory (left) obtained from interference microscopy (right). The microparticles were (A) trapped between the electrodes, (B) oscillating between the electrode tips, or (C) attached to the electrode edge. The trajectory of sample microparticle 1 (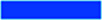) and sample microparticle 2 (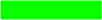). Scale bar = 100 μm.

**Fig. 3:**
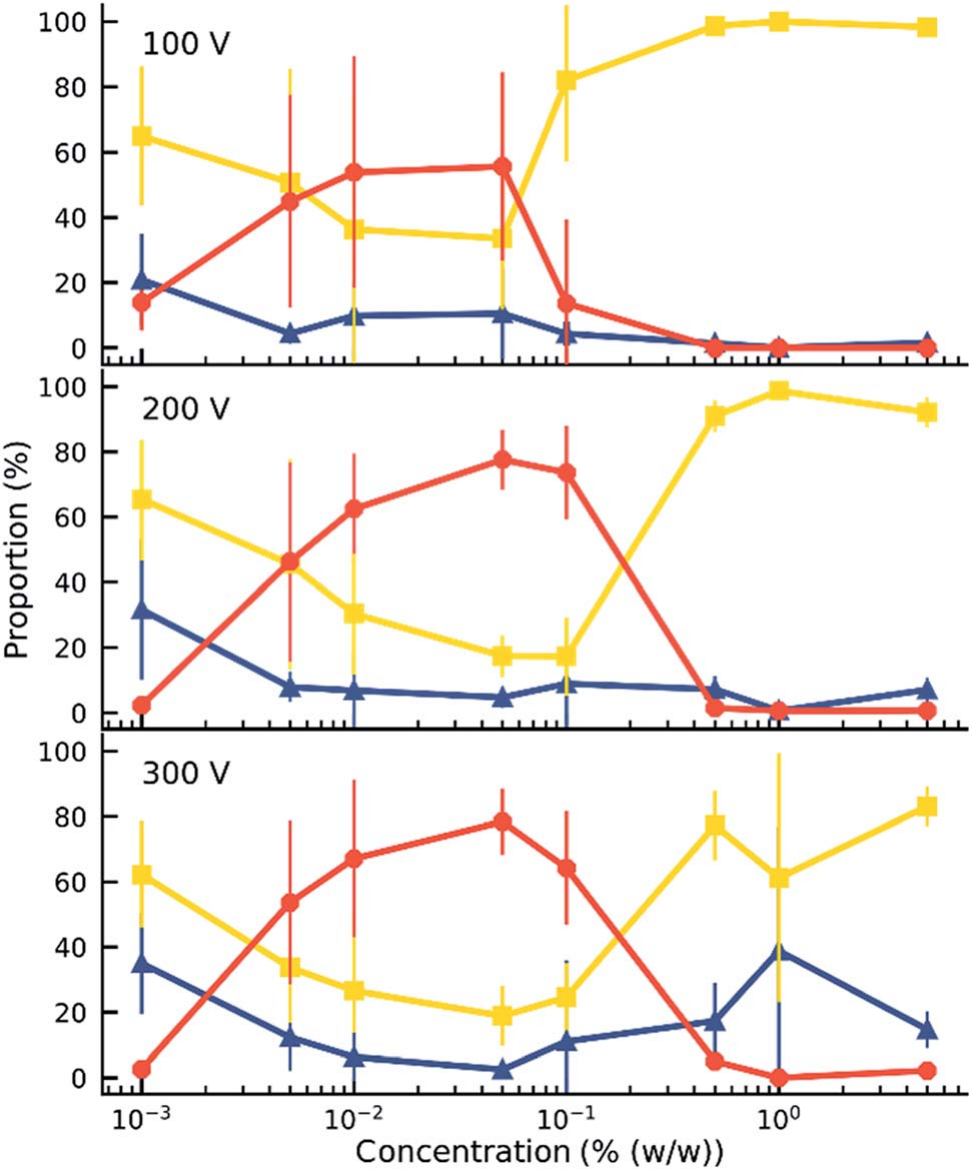
Proportion of trapped (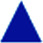), oscillating (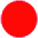), and attached (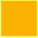) microparticles in a sample according to the surfactant concentration and applied voltage. The solid lines are guides to the eye. Error bars indicate standard deviation.

When an electric field was applied to the dispersed microparticles in oil with surfactant using sawtooth electrodes, the microparticles exhibited one of three motion patterns regarding their position and displacement as follows: ‘trapped’, when the microparticle remained between the electrodes without touching them Fig. 2A; ‘oscillating’, when the microparticles moved periodically between the electrodes Fig. 2B; and ‘attached’, when the microparticles remained in close contact with one of the electrodes Fig. 2C. The microparticles in the same sample did not all exist in the same state and, on occasion, a microparticle would switch from one state to another. However, the proportion of microparticles in a certain state within a sample remained approximately constant depending on the surfactant concentration and applied voltage (Fig. 3). The oscillating microparticles were most abundant when the surfactant concentration was approximately 0.05% (w/w), while most microparticles were in the attached state when the surfactant concentration was <0.005% (w/w) or >0.5% (w/w).

**Fig. 4:**
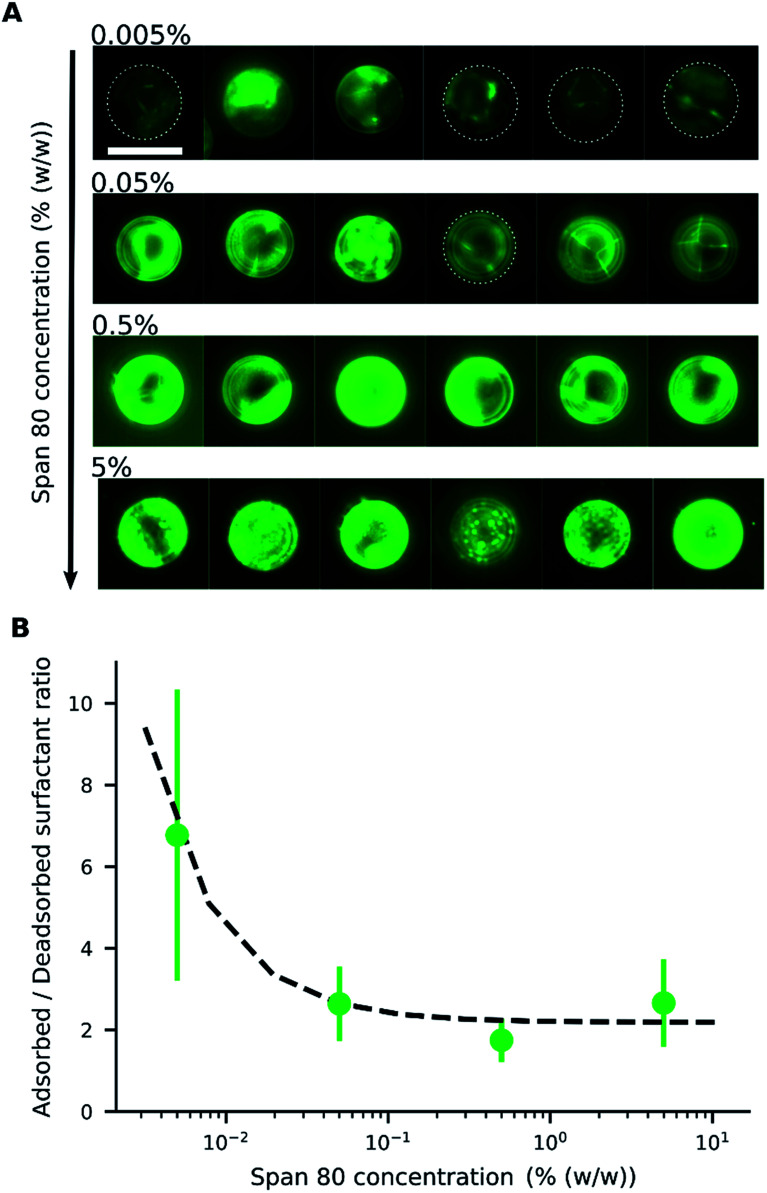
Adsorption of reverse micelles loaded with fluorescein on the microparticles. (A) Fluorescence images of microparticles with different concentrations of Span 80; images in the same row were taken from the same sample. Dotted lines delimit the contour of microparticles with low fluorescence. Scale bar = 20 μm. (B) Ratio of surfactant adsorbed on the microparticles and dispersed in the medium according to surfactant concentration as measured by fluorescence (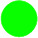) and according to the Langmuir adsorption model, fitted to the experimental points (---).

To investigate the role of the surfactant in the mechanism of motion, reverse micelles of Span 80 with a loaded fluorescent dye were used to observe the location of the surfactant within the dispersion. The surfactant reverse micelles with fluorescein were initially dispersed homogeneously, but they subsequently adsorbed on the microparticle surfaces (Fig. 4A). The adsorption was not homogeneous among the microparticles and varied depending on the final concentration of surfactant (Fig. 4A). Fluorescein sodium is a salt and does not dissolve in oil or adsorb directly on the surface of the polystyrene microparticles; therefore, the average fluorescence intensity of the beads and the background was assumed to be proportional to the local concentration of surfactant. We assumed for simplicity that the adsorption of reverse micelles loaded with fluorescein on the microparticle surface does not significantly enhance the self-quenching of fluorescein, which could occur when fluorescein concentration increases,^38^ causing an underestimation of adsorption at higher surfactant concentrations.

By measuring the fluorescence ratio between the microparticles and oil, the ratio of the surfactant on the microparticle surface to that in the oil was determined (Fig. 4B). At low surfactant concentrations, the ratio of adsorbed surfactant on the desorbed surfactant was high, but the surface of the microparticles became saturated with a constant ratio as the surfactant concentration increased. The ratio of absorbed to desorbed surfactant, derived from the Langmuir adsorption model,^39^ is given by eqn (1):1
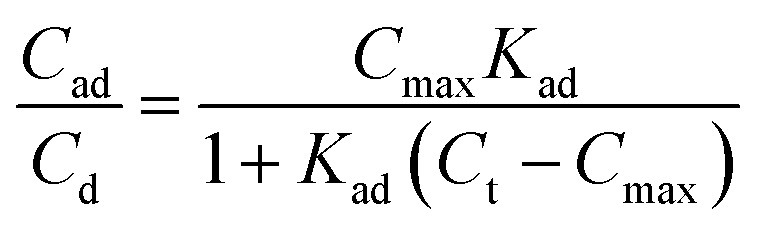
where *C*_ad_ is the concentration of adsorbed surfactant on the microparticle surface; *C*_d_ is the surfactant concentration dissolved in the medium; *C*_max_ is the maximum possible concentration of adsorbed surfactant; *K*_ad_ is the adsorption equilibrium constant; and *C*_t_ is the total surfactant concentration (see ESI Discussion 1†). To fit the experimental data, a baseline was used (Fig. 4B).

**Fig. 5:**
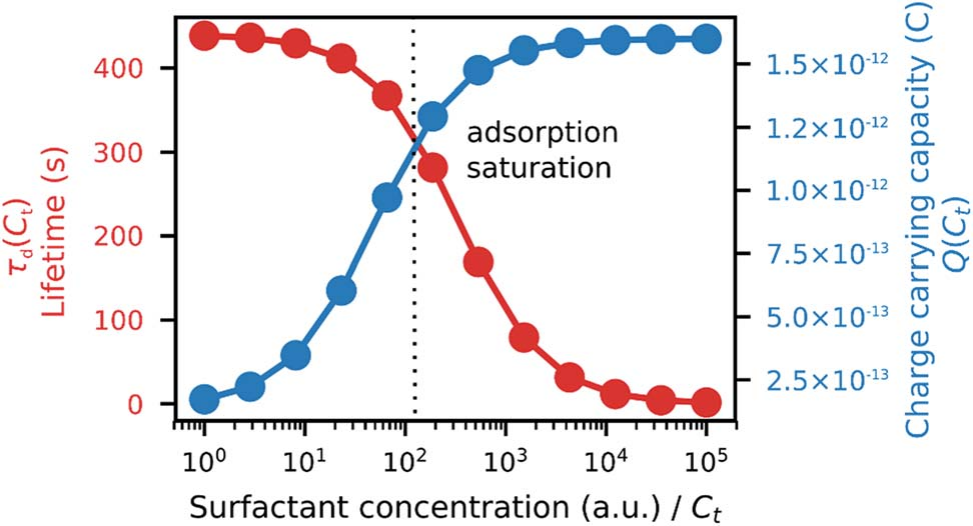
Model on how surfactant concentration affects the parameters used in the simulation of microparticle trajectory: charge carrying capacity (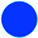) and charge relaxation lifetime (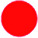). Parameters used in the simulation: *q*_ar_ ≈ 1.48 × 10^−12^; *K*_ad_ ≈ 2.74 × 10^−2^; *σ*_0_ ≈ 8.88 × 10^−3^; *q*_s_ ≈ 4.44 × 10^−5^.

As the reverse micelles can stabilize charges in apolar media,^22,25,30,40,41^ two properties of the suspension depend on surfactant concentration: the microparticle charge and medium conductivity. These properties depend mainly on the local surfactant concentration; that is, the microparticle charge is limited by the amount of surfactant adsorbed, *C*_ad_, while the medium conductivity, *σ*, is limited by the amount of surfactant in the medium, *C*_d_. The charge ceiling of the microparticle was defined as the charge carrying capacity *Q*(*C*_t_), as in eqn (2) below:2
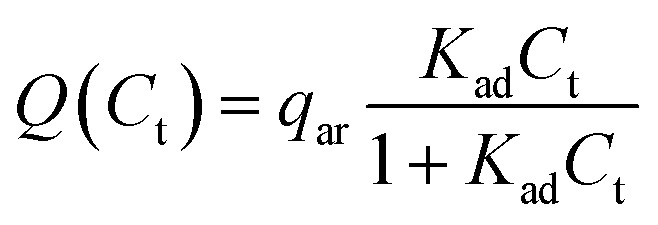
where *q*_ar_ is the charge per area per adsorbed surfactant unit (see Fig. 5 and ESI Discussion 1†).

The conductivity of a medium is proportional to the surfactant concentration in it (observed for Span 80 in hexane^28^ and isopar-L^41^), which can be defined in terms of the charge relaxation law: if a charged particle is suspended in a fluid with uniform conductivity, *σ*, and dielectric constant, *ε*_m_, its charge decays with a time constant *τ*_d_ = *ε*_m_/*σ*.^42^ Therefore, considering the surfactant effects on the medium conductivity, eqn (3) can be obtained:3
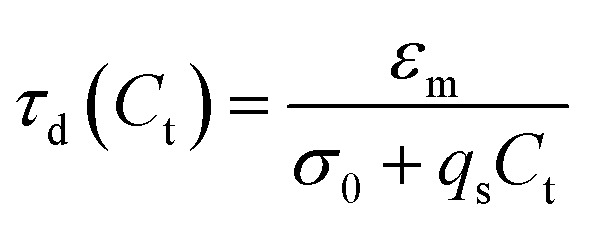
where *σ*_0_ is the conductivity of pure liquid paraffin, *q*_s_ is the rate of conductivity increase per surfactant unit in the medium, and *C*_d_ ≈ *C*_t_ for low microparticle concentrations (Fig. 5).

**Fig. 6:**
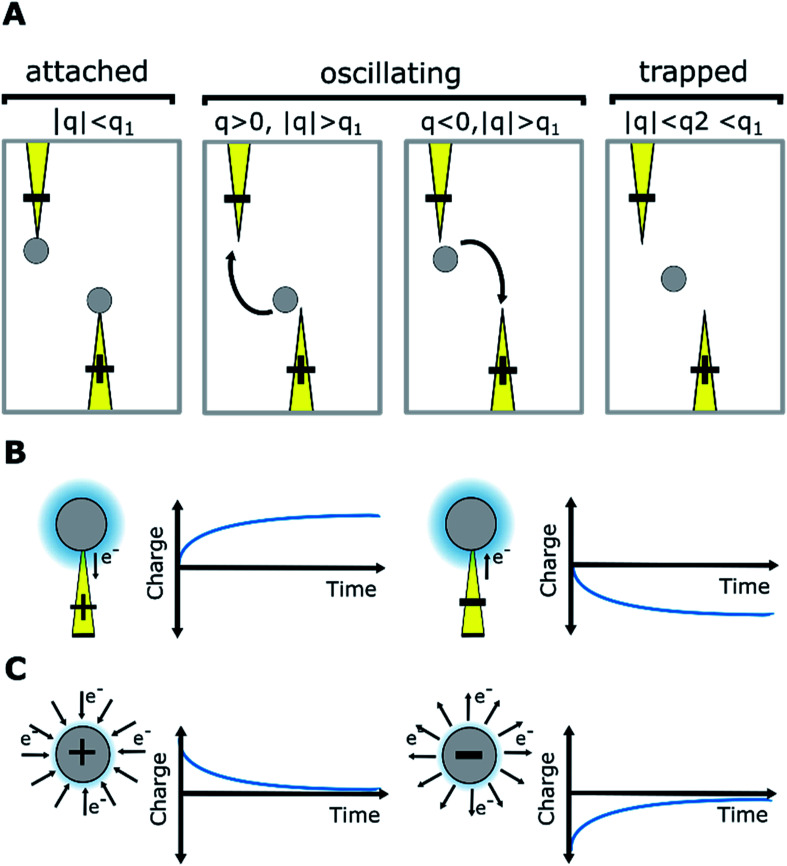
(A) Microparticle trajectory indicates the sign and magnitude of charge; *q* is the particle instant charge, *q*_1_ and *q*_2_ are charge thresholds. (B) Microparticle charging. (C) Microparticle charge relaxation.

From the experimental trajectories of the microparticles, as those shown in Fig. 2, it is possible to guess the sign and magnitude of the microparticle charge (Fig. 6A). For example, a microparticle attaches to the electrodes when its instant charge *q* is smaller than a charge threshold *q*_1_, *q*_1_ such that the electric force is smaller than the dielectric and viscous forces. If the charge is above this threshold, the particles migrate to the electrodes of opposite charge. However, when the particle is migrating, it can eventually be trapped between the electrodes, where the dielectric force is null, if the electric force is smaller than the viscous force, that is, if the microparticle instant charge is smaller than *q*_2_, *q*_2_ being smaller than *q*_1_. The oscillating microparticles show the microparticle can exchange charge when they touch the electrodes, while trapped microparticles show they can lose charge when they are not touching the electrode, as defined by the charge relaxation law (Fig. 6B). Eqn (4) was used to describe the change in charge after a microparticle touches an electrode, which is derived from the charging of a sphere by a unipolar current^42–46^ (see ESI Discussion 2†):4
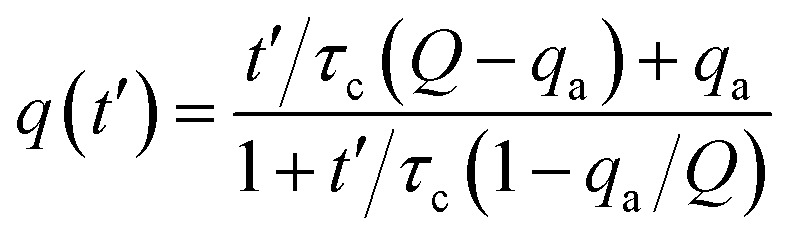
where *t*′ is the time after a particle attaches to the electrode; *q*(*t*′) is the microparticle charge at time *t*′; *τ*_c_ is a charging rate parameter; and *q*_a_ is the charge of the particle when the particle attaches to the electrode. In eqn (4), the charge *q*(*t*′) approaches *Q* as *t*′ → ∞ and the rate of charging decreases as the charge approaches this limit. When the microparticles are not contacting the electrodes, their charge decreases exponentially, as described in eqn (5):

5 *q*(*t*′′) = *q*_d_e^−*t*′′/*τ*_d_^

where *t*′′ is the time after a particle detaches from the electrode; *τ*_d_ is the charge relaxation lifetime; and *q*_d_ is the charge of the particle when the particle detaches from the electrode.

In our model, *Q* and *τ*_d_ are functions of the surfactant concentration (eqn (2) and (3)). In that sense, the surfactant in an apolar medium promotes opposing mechanisms of particle charging and charge relaxation, both of which depend on concentration.

**Fig. 7:**
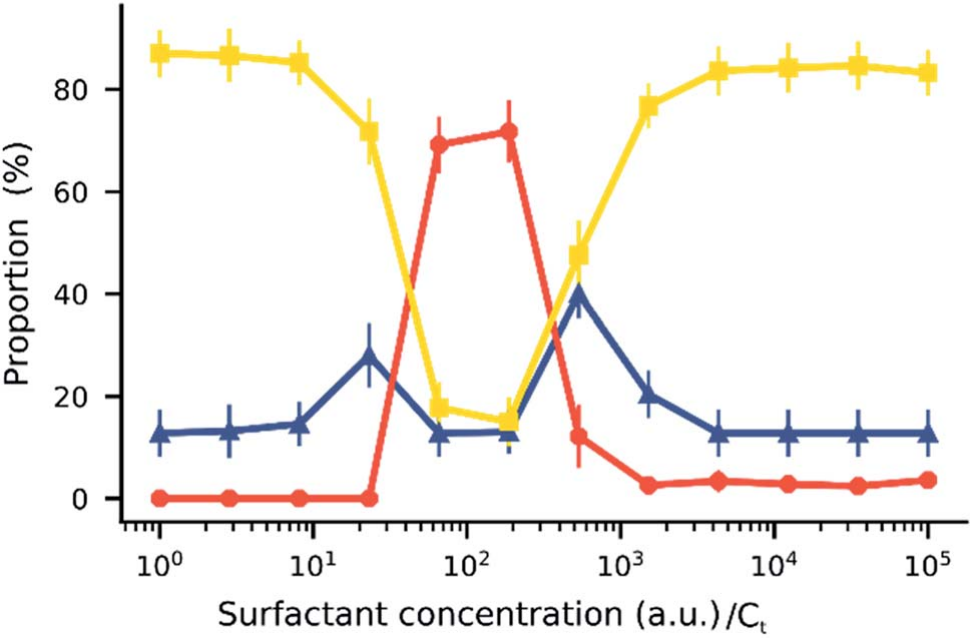
Simulated distribution of trapped (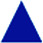), oscillating (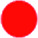), and attached (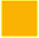) microparticles according to the surfactant concentration. Each point represents the average of 10 sets of 50 trajectories each. Error bars indicate standard deviation.

Using eqn (2)–(5) and parameters estimated from the literature (see ESI Note 1†), the trajectories of 500 microparticles were simulated while varying the charge carrying capacity and charge relaxation lifetime, while emulating increasing surfactant concentrations (Fig. 7). To simulate the particle trajectory, the electric and dielectric fields were simulated using finite elements software. The electric force 
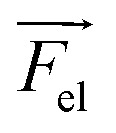
,6
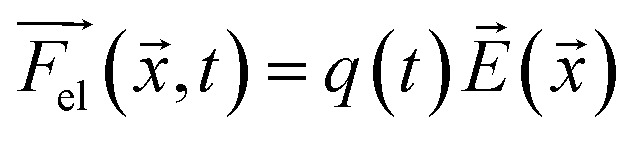
depends on the particle charge *q*(*t*), where *t* is time and 
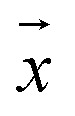
 is the microparticle position. The dielectric force 
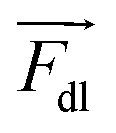
, which originates from the interaction of the inhomogeneous electric field and particle dipole, can be given as follows:7
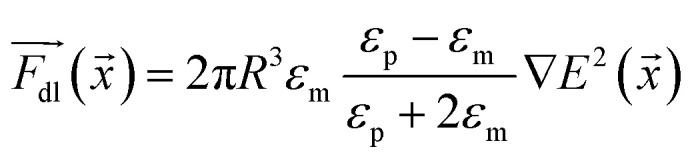


It is proportional to *R*^3^, where *R* is the particle radius and depends on *ε*_m_ and *ε*_p_, the dielectric constants of the medium and particle, respectively. In our system, *ε*_m_ > *ε*_p_, which means the dielectric force attracts the microparticles towards the electrode tips, where the electric field is more divergent. Thereafter, the overdamped equation of motion can be constructed as follows:8
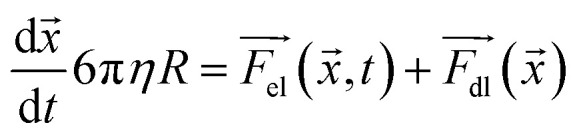
where the left side expresses the viscous force (Stokes drag force) predominant in microenvironments.^9^

During the simulation, small variations in the initial conditions—such as initial position, velocity, and charge—yielded different trajectories (see ESI Note 2†). To account for this observation, the simulations were performed using 500 initial conditions that were later randomly split into 10 sets; the results are shown in Fig. 7. The simulation suggests that the trajectory of a microparticle depends on its initial conditions, although the proportion of each motion pattern is approximately constant and defined by surfactant concentration.

**Fig. 8:**
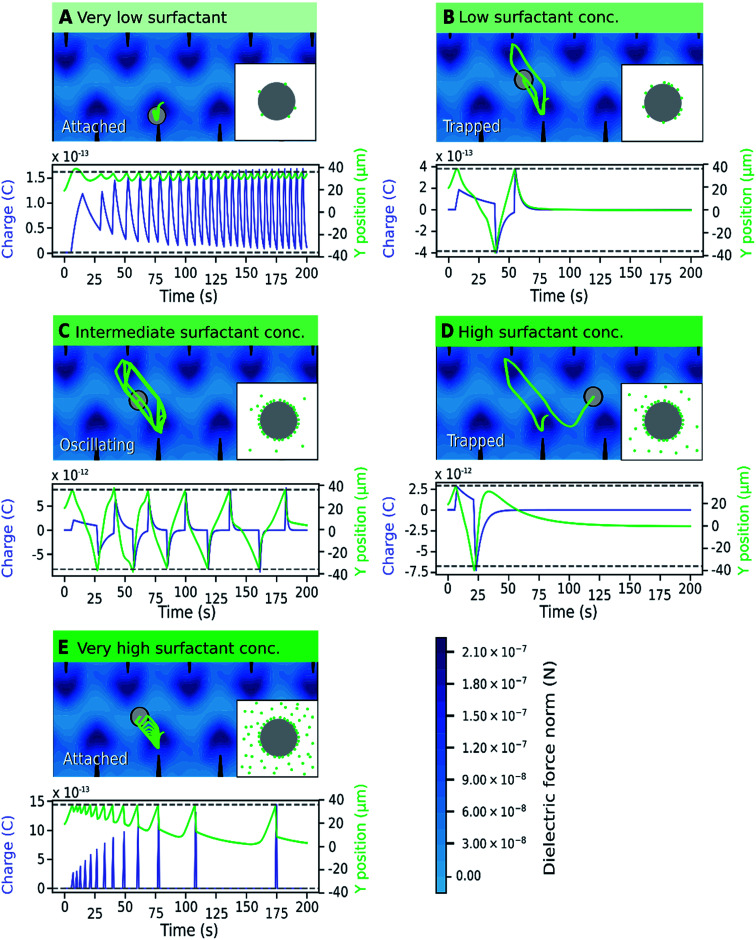
Simulated trajectory of a microparticle at various surfactant concentrations demonstrating the effect of changing the parameters charge carrying capacity (*Q*) and charge relaxation lifetime (*τ*_d_). The underplot shows the microparticle position (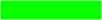) and charge (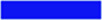) as a function of time in relation to the tip of the electrodes (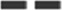). (A) *Q* ≈ 1.75 × 10^−13^ C, *τ*_d_ ≈ 437.81 s; (B) *Q* ≈ 8.44 × 10^−13^ C, *τ*_d_ ≈ 357.11 s; (C) *Q* ≈ 1.26 × 10^−12^ C, *τ*_d_ ≈ 239.90 s; (D) *Q* ≈ 1.56 × 10^−12^ C, *τ*_d_ ≈ 37.37 s; (E) *Q* ≈ 1.59 × 10^−12^ C, *τ*_d_ ≈ 0.87 s.

The relationship between particle trajectory and charge was examined to better understand the origin of the different motion patterns. A representative simulation of a particle trajectory is shown in Fig. 8, with the model of local concentration of surfactant aggregates in the insets. The surfactant concentration changes the microparticle charge carrying capacity and relaxation lifetime, which changes the balance between the dielectric force (field shown in Fig. 8) and electric force. The dielectric force pushes the particle towards the electrode tips whereas the electric force depends on the position and particle charge, which changes dynamically due to charging and charge relaxation mechanisms enabled by the surfactant. When a microparticle is charged, it migrates towards the electrode with the opposite charge. Generally, attached microparticles were observed when the dielectric force was dominant and forced them close to the electrode tips. This occurred at very low and very high surfactant concentrations. At very low concentrations, the surfactant concentration is low both on the microparticle and in the medium and the microparticle charge is low. At very high surfactant concentrations, charge relaxation is rapid due to the increased amount of surfactant in the medium and microparticles have a low average charge. At marginally low and marginally high surfactant concentrations, trapped microparticles were more common. Trapped microparticles were observed when the charge decayed while the microparticles were close to the stable point between the electrodes, where the dielectric force is null. Oscillating microparticles were most common at intermediate surfactant concentrations, when the charge carrying capacity and charge relaxation lifetime were high. At this concentration range, the microparticle has sufficient charge to reach the opposite electrode without becoming trapped at the stable point. When the particle reaches the opposite electrode, it is charged with the opposite charge and thus the cycle is restarted.

A comparison of Fig. 7 and 3 demonstrates the newly developed model succeeds in explaining the enhanced oscillation or trapping at certain surfactant concentration and the concentration thresholds. It should be cautioned that the model is two-dimensional, estimating forces on the microparticles that are not equivalent to what would be expected in actual experiments. For instance, in Fig. 8 the oscillating frequencies are significantly lower than those observed experimentally (Fig. 2). Furthermore, changes in other parameters of the system were not considered, but are expected upon increasing surfactant addition, such as the dielectric constant^47^ and viscosity.^48^

## Conclusions

Surfactants change the electrokinetics of microparticles by enabling their charging and discharging in an electric field. Varying surfactant concentration changes the ratio of surfactant adsorbed on the microparticle to that in the medium, which charges the microparticles when in contact with electrodes and discharges the microparticle in the medium. The change in charge modifies the balance between the electric, dielectric, and viscous forces, creating a dynamic system wherein the microparticles display different motion patterns depending on surfactant concentration. This indicates that a static, inhomogeneous electric field is a versatile tool for particle control and surfactants can act as a mediator to integrate micromachines and digital devices. The further understating of microparticle charging mediated by surfactants may allow for the improved self-assembly of microstructures,^49^ motion of microrobots,^50^ and individual microparticle control^51^ by designing novel electrode geometries. The newly developed model can also provide a rationale for the syntheses of novel surfactants for use in electric sensitive colloids^52^ by tuning surfactant adsorption and charged aggregate formation.

## Conflicts of interest

There are no conflicts to declare.

## Supplementary Material

RA-010-D0RA00703J-s001

RA-010-D0RA00703J-s002

RA-010-D0RA00703J-s003

RA-010-D0RA00703J-s004

RA-010-D0RA00703J-s005

RA-010-D0RA00703J-s006

RA-010-D0RA00703J-s007
