# Supplementary material for: Surfactant concentration modulates the motion and placement of microparticles in an inhomogeneous electric field
Source: RSC Adv. 2020 Mar 2;10(15):8895–904. doi: 10.1039/d0ra00703j (PMC9050010; doi:10.1039/d0ra00703j)
Supplement: RA-010-D0RA00703J-s007 [file RA-010-D0RA00703J-s007.pdf]

Electronic Supplementary Material (ESI) for RSC Advances.
This journal is © The Royal Society of Chemistry 2020

Supplementary Materials for

Surfactant concentration modulates the motion and placement of microparticles in an inhomogeneous electric field

Marcos Masukawa[a] , Masayuki Hayakawa [b,c] and Masahiro Takinoue [*ab]

[a]Department of Computer Science, Tokyo Institute of Technology, 4259 Nagatsuta-cho, Midori-ku, Yokohama, Kanagawa, 226-8502, Japan.

[b]Department of Computational Intelligence and Systems Science, School of Computing, Tokyo Institute of Technology, 4259 Nagatsuta-cho, Midori-ku, Yokohama, Kanagawa226-8502, Japan.

[c]RIKEN Center for Biosystems Dynamics Research, Kobe, Hyogo 650-0047, Japan.

*Correspondance to M.T. E-mail: takinoue@c.titech.ac.jp.

This PDF file includes:

Supplementary Methods

Figs. S1 to S3

Supplementary Discussion

Figs. S4 to S5

Supplementary Notes

Fig. S6

Other Supplementary Materials for this manuscript includes the following:

Movies S1 to S6

available at https://takinouelab.github.io/MasukawaTakinoue2019/

Supplementary Methods

1) Device design and experiment assembly

The electrodes were designed using the software Rhinoceros (Robert McNeel & Associates, v4.0) and consisted of interdigitated electrodes with a saw-tooth edge, as shown in Fig. S1. This arrangement was used to maximize the number of electrodes on the slide. Copper tape and conductive adhesive were used to connect the electrodes to a direct current source. Twelve microliters of the sample were pipetted in the well, bubbles were removed with a blower, and the microparticles were observed from bottom to top of the slide using an inverted microscope.

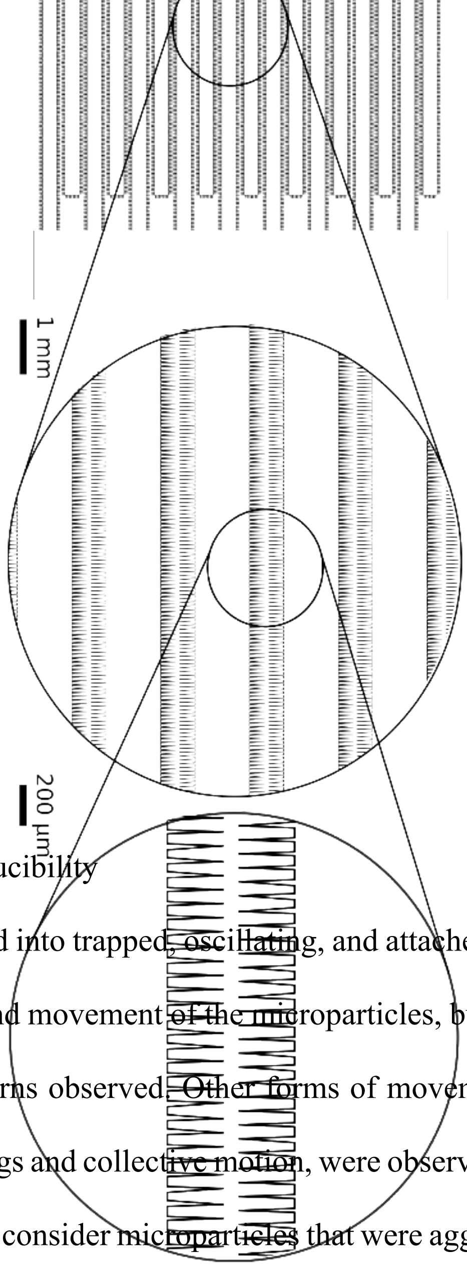

Figure S1. – Electrode design.

2) Microparticle classification and reproducibility

The microparticles were visually classified into trapped, oscillating, and attached states. The states were named based on the position and movement of the microparticles, but do not comprehensively describe all the motion patterns observed. Other forms of movement and organization of the microparticles, such as strings and collective motion, were observed when the microparticles were aggregated. We did not consider microparticles that were aggregated; therefore, we used a low concentration of microparticles to minimize particle-particle interaction. Only microparticles that were in the area of interest shown in Fig. S2 were considered. In some cases, microparticles would change from one state to the other; in this situation, the microparticle was not reclassified. Additionally, the behavior of the microparticles was highly dependent on the preparation method, that is, a sample that was only vortexed would have a different distribution of states compared to a sample that also was sonicated.

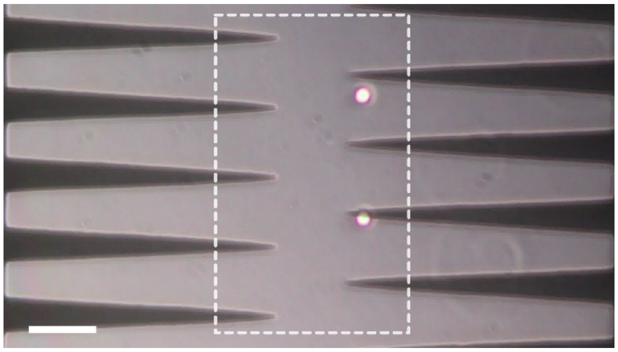

Figure S1 – Area of interest where microparticles where classified. Scale bar = 70 μm.

3) Measurement of background fluorescence

To calculate the ratio between the fluorescence of microparticles and medium, we took fluorescent micrographs of 11 areas sized 832 μm 702 × 832 μm of a sample in a 0.2 mm thick polydimethylsiloxane PDMS gasket. The average fluorescence intensity (sum of pixel intensity of region of interest divided by the number of pixels) of the microparticles was measured. Then, image analysis software was used to exclude the microparticles and the average fluorescence intensity of the background was measured. The background was mostly homogeneous, but surfactant aggregates with fluorescein could be observed at higher surfactant concentrations (Fig. S3).

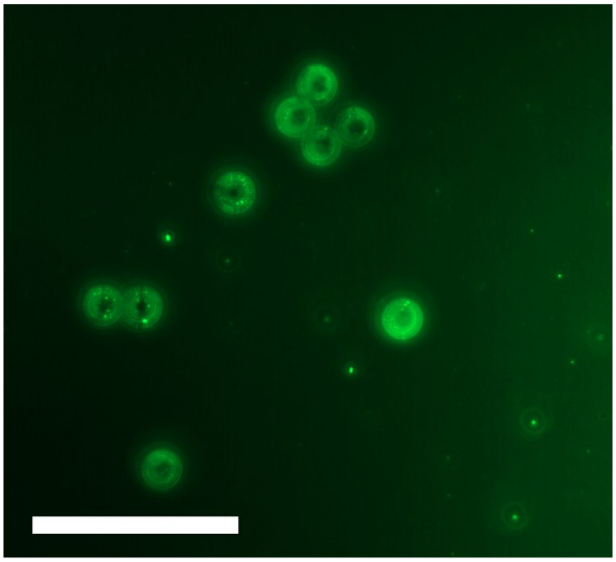

Figure S3 – Particles covered with Span 80/fluorescein aggregates at the final Span 80 concentration of 5% (w/w). Scale bar = 200 μm.

Supplementary Discussion

1) Langmuir adsorption model

The ratio of adsorbed and desorbed surfactant based on the surfactant concentration was fit with an equation derived from the Langmuir adsorption model[1]. The model assumes the surface of the microparticle is formed by discrete adsorption sites that the adsorbates, in this case the surfactant aggregates, can occupy (Fig. S4). The model considers that the adsorbates do not interact with each other and do not move, that the surface is homogeneous, and that the adsorption sites can only be occupied by one adsorbate at the time. We used this model as a first approximation with which to relate surfactant concentration and surfactant adsorbed. The model treats the adsorption as a reversible chemical reaction and gives the proportion of occupied sites in relation to the concentration of the adsorbent. In Eq. S1, θ_A corresponds to the fraction of the surface covered by the reverse micelles, K_{ad} is the adsorption equilibrium constant, and C_t is the surfactant concentration[138].

$$\theta_A = \frac{K_{ad}C_t}{1 + K_{ad}C_t} \qquad (S1)$$

If it is assumed the maximum amount of charge the microparticle can have, Q, is proportional to the amount of surfactant on the surface of the microparticle, we obtain Eq. S2, as given below, where q_{ar} is a constant that relates units of surfactants on the surface of the microparticle and charge:

$$Q(C_t) = q_{ar} \frac{K_{ad}\, C_t}{1 + K_{ad}\, C_t} \qquad (S2)$$

To calculate the ratio of surfactant adsorbed on the microparticle and the surfactant in the medium, let C_{max} be the maximum possible concentration of adsorbed surfactant and C_{ad} the concentration of adsorbed surfactant. Therefore, the ratio of adsorbed to desorbed surfactant is $C_{ad}/(\, C_t - C_{ad})$. If we consider that the adsorbed surfactant is proportional to the occupied sites at the surface of the microparticle, we have Eq. S3, where $\alpha = C_{max}K_{ad}$ and $\beta\ = 1 - \alpha$ are constants.

$$C_{ad} = \frac{C_{max}K_{ad}C_t}{1 + K_{ad}C_t}$$

$$\frac{C_{ad}}{C_t - C_{ad}} = \frac{\dfrac{C_{max}K_{ad}C_t}{1 + K_{ad}C_t}}{C_t - \dfrac{C_{max}K_{ad}C_t}{1 + K_{ad}C_t}} = \frac{C_{max}K_{ad}}{1 + K_{ad}(C_t - C_{max})}$$

$$\frac{C_{\mathrm{ad}}}{C_d} = \frac{\alpha}{\beta + K_{\mathrm{ad}}C_{\mathrm{t}}} = \frac{1}{\dfrac{\beta}{\alpha} + \dfrac{C_{\mathrm{t}}}{C_{\mathrm{m}}}} \qquad (S3)$$

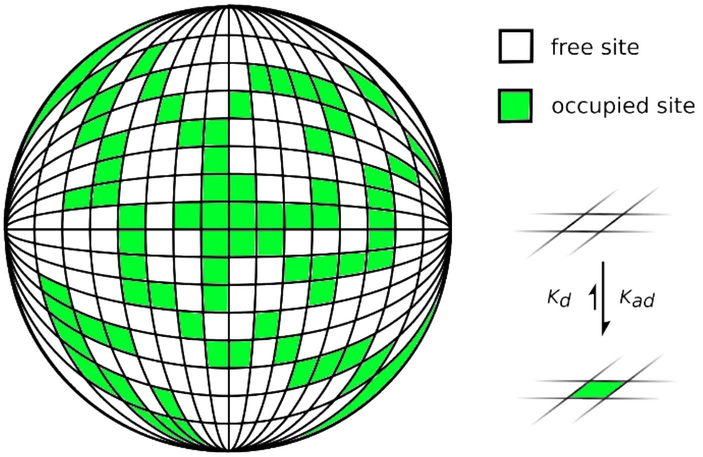

Figure S4 – Langmuir adsorption model on the surface of a sphere.

2) Models of microparticle charging

Smith and McDonald[2] developed a theoretical model for charging of dust microparticles by ions. Based on their work, we establish an analogy to the charging of microparticles by charged reverse micelles, which provides an equation for the charging of microparticles mediated by surfactant. The original model described a system where dust microparticles are suspended in air that contains ions. When an electric field is applied, there is a flow of ions that charges the suspended microparticles[3,4]. In our system, the analogous insulating media is an apolar liquid instead of air, the ions are the charged reverse micelles, and the dust microparticles are the polystyrene microparticles. According to this model, the rate of charging is given by Eq. S4, where q is the instantaneous charge of the microparticle, Q is the saturation charge (or charge carrying capacity), N_0 is the charge density in the medium, z is the electrical mobility of the charges and ϵ_m is the dielectric constant of the medium. $4\epsilon_m/(N_0 zQ)$ is a parameter of how fast the charging occurs; we denoted it τ_c.

$$\frac{dq}{dt} = \frac{N_0 zQ}{4\epsilon_m}\left(1 - \frac{q}{Q}\right)^2$$

$$\frac{dq}{dt} = \frac{Q}{\tau_c}\left(1 - \frac{q}{Q}\right)^2$$

$$\frac{d(q/Q)}{d(t/\tau_c)} = \left(1 - \frac{q}{Q}\right)^2 \qquad (S4)$$

However, Dascalescu et al.[5] noted that this charging rate is precise for microparticles charged by a current produced by a uniform electric field, which is not the case in our system. Taking that into consideration, we will assume that it remains valid as a qualitative description. In this model, the greater the charge the microparticle has, the slower it will charge $dq/dt \rightarrow 0$ as $q/Q \rightarrow 1$; therefore, it cannot have a charge greater than the charge carrying capacity Q ($q \rightarrow Q$ as $t \rightarrow \infty$). To obtain the charge of a microparticle at time t' that had charge q_a when it touched the electrode at time t', we can make a variable substitution, use the chain rule and integrate Eq. S4, resulting in the charging Eq. S5. For simplicity, we considered the initial time as zero. The effect of the parameters Q, $q(0)$, τ_c on charging is illustrated on Fig. S5.

$$\int_{q_a/Q}^{q/Q} \frac{dx}{(1 - x)^2} = \int_0^{t/\tau_i} dy$$

$$\left.\frac{1}{1 - x}\right|_{q_a/Q}^{q/Q} = \frac{t}{\tau_c}$$

$$q(t) = \frac{\frac{t}{\tau_c}(Q - q_a) + q_a}{1 + \frac{t}{\tau_c}\left(1 - \frac{q_a}{Q}\right)} \qquad (S5)$$

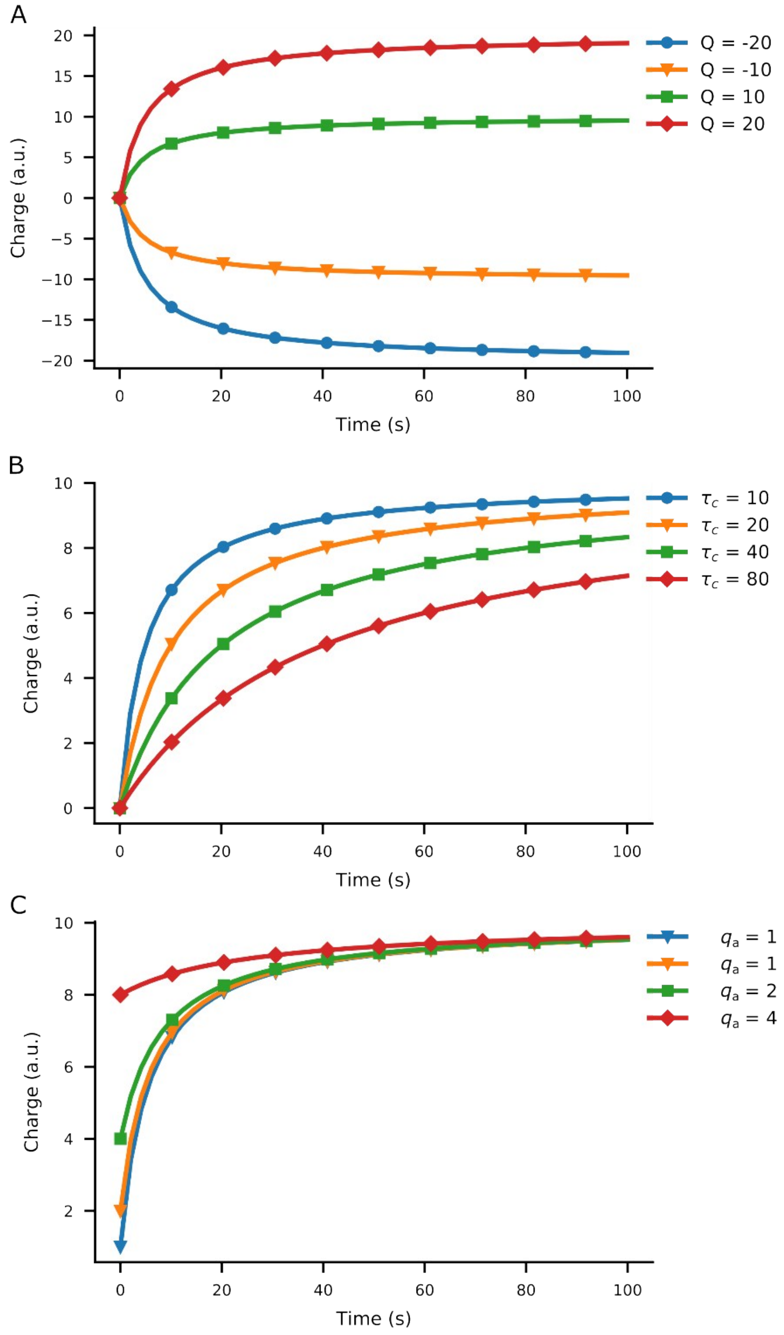

Figure S5 – (A) Charging with different charging capacities Q, $q_a = 0$, $\tau_c = 5$. (B) Charging at different rates τ_c, $Q = 10$, $q_a = 0$. (C) Charging at different initial charges q_a, $Q = 10$, and $\tau_c = 5$.

Supplementary Notes

1) Choice of simulation parameters

In our model, the microparticles are charged when they are in contact with the electrode, according to Eq. 4, and they are discharged due to charge relaxation according to Eq. 5. Although this mechanism is evidenced by the trajectory of the microparticles, it is not possible to obtain the charging and discharging parameters concomitantly from the analysis of the trajectory. To estimate the charge carrying capacity, we examined the order of the magnitude of the microparticle charges in apolar colloids with surfactants and the charge relaxation time of similar media. For instance, Espinosa et al.[6] studied the system of sub-micrometre PMMA microparticles in hexane with Span 85 and observed that they have charges in the order of tens of elementary charges. Microparticles of 20 μm would have a surface area 3 to 4 orders of magnitude larger, meaning that the average charge of a microparticle would be due to elementary changes of 10^4 to 10^5. Hsu et al.[7] observed that 780 nm PMMA microparticles in dodecane with dioctyl sulfosuccinate sodium salt (AOT) have 290 ± 30 elementary charges and proposes a model that constrains the maximum number of charges in the microparticles to 10^6. For 20 μm diameter microparticles, that would mean microparticles with charges in the order 10^5 elementary charges and limited to approximately 10^9 charges. In our simulation, the charge carrying capacity varies between $\sim 10^6$ and $\sim 10^7$ elementary charges, depending on the surfactant concentration. Along these values, the charge relaxation time, τ_d, was valued between ~ 450 s and ~ 1 s. For comparison, mica, which is used as an electric insulator, has a relaxation time of 51,000 s and corn oil, which is a weakly conductive oil, has a τ_d of 0.55 s[8].

2) Choice of initial conditions

In the experiments, the microparticles were uniformly distributed in the sample; therefore, we assumed random initial positions in the simulation. The initial velocity had a random orientation and a module following a Gaussian distribution of average zero and variance of 0.05 μm/s. For the initial charge of the microparticles, we considered the magnitude of the charges observed by Espinosa et al.[6] and Hsu et al.[7]. In our simulation, the microparticles started with a Gaussian distribution of charges with average charge zero and variance of 10^3 elementary charges, which is equivalent to approximately 16 fC.

3) Microparticle classification in the simulation

The simulations lasted 200 s and the first and last 40 s were discarded. To classify the microparticles, we counted the number of times the microparticle left each electrode. If the microparticle did not leave the electrodes at any time and its position was between the electrodes (that is, it was not ejected from the simulation area), then it was considered trapped. If the microparticle left one electrode more than the other as defined by a tolerance then it was considered attached, and if it touched both electrodes for approximately the same number of times as defined by a tolerance parameter it was considered to be oscillating (see flowchart in Fig. S6). In our simulation, we used a tolerance value of 0.2.

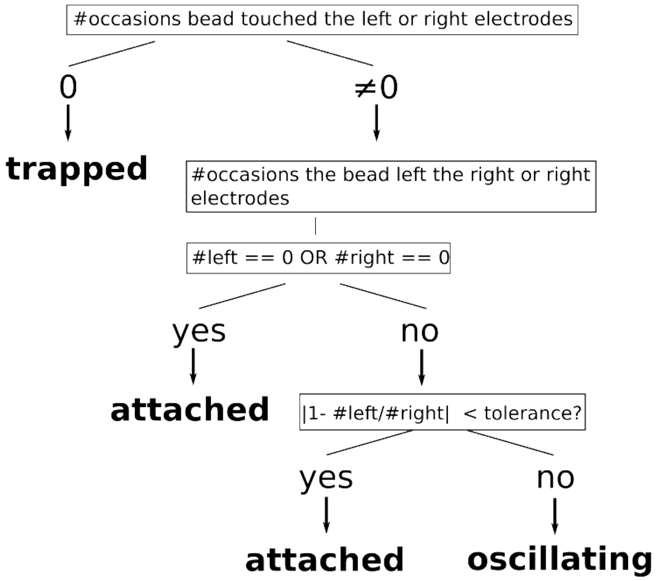

Figure S6 – Algorithm for microparticle classification used in the simulation.

Supplementary Videos

Videos available at

https://takinouelab.github.io/MasukawaTakinoue2019/

Movie S1

Adsorption of Span 80 aggregates containing fluorescein on the microparticles does not hinder oscillation in the electric field produced by the sawtooth electrodes.

Movie S2

Particle trajectory simulation at a very low concentration of surfactant.

Movie S3

Particle trajectory simulation at a low concentration of surfactant.

Movie S4

Particle trajectory simulation at an intermediate concentration of surfactant.

Movie S5

Particle trajectory simulation at a high concentration of surfactant.

Movie S5

Particle trajectory simulation at a very high concentration of surfactant.

Supplementary material references

1 I. Langmuir, J. Am. Chem. Soc., 1932, 54, 2798–2832.

2 W. B. Smith and J. R. McDonald, J. air Pollut. Control Assoc., 1975, 25, 168–172.

3 M. Pauthenier and M. Moreau-Hanot, J. Phys. Radium, 1932, 3, 590–613.

4 H. J. White, in Industrial electrostatic precipitation, Addison-Wesley, 1963.

5 L. Dascalescu, D. Rafiroiu, A. Samuila and R. Tobazeon, in Industry Applications Conference, 1995. Thirtieth IAS Annual Meeting, IAS'95., Conference Record of the 1995 IEEE, 1995, vol. 2, pp. 1229–1234.

6 C. E. Espinosa, Q. Guo, V. Singh and S. H. Behrens, Langmuir, 2010, 26, 16941–16948.

7 M. F. Hsu, E. R. Dufresne and D. A. Weitz, Langmuir, 2005, 21, 4881–4887.

8 H. A. Haus and J. R. Melcher, Electromagnetic fields and energy, Prentice Hall, 1989.
